# Extra-Oral Three-Dimensional (3D) Scanning Evaluation of Three Different Impression Materials—An In Vitro Study

**DOI:** 10.3390/polym14173678

**Published:** 2022-09-05

**Authors:** Eugen S. Bud, Vlad I. Bocanet, Mircea H. Muntean, Alexandru Vlasa, Mariana Păcurar, Irina Nicoleta Zetu, Bianca I. Soporan, Anamaria Bud

**Affiliations:** 1 Faculty of Dental Medicine, University of Medicine and Pharmacy, Science and Technology George Emil Palade, 540139 Târgu-Mureș, Romania; 2 Department of Industrial Engineering, Faculty of Industrial Engineering, Robotics and Production Management, Technical University of Cluj-Napoca, 400144 Cluj-Napoca, Romania; 3 Department of Orthodontics and Dento-Facial Orthopedics, Grigore T. Popa University of Medicine and Pharmacy, 700115 Iași, Romania

**Keywords:** three-dimensional impression, alginate impression, silicone impression

## Abstract

Impression materials are used to record and reproduce the exact morphology of the patient’s oral cavity. The dimensional stability of a material is its ability to maintain the accuracy of recording the details of the oral cavity for a longer period of time, including the time during imprinting and immediately after. The aim of this study was to evaluate the accuracy of three different impression materials commonly used in the dental practice with the aid of an extra-oral three-dimensional (3D) scanner using an in vitro analysis. A typodont tooth model of the maxillary dental arch and mandibular dental arch, containing 16 permanent teeth, was used for evaluation. With the aid of three different impression materials, this model was imprinted fifteen times, resulting in fifteen different plaster models. A capsule extra-oral scanner device was used to digitalize the models and the same device was later used to align, compare, and measure scanned model surfaces. After performing the Kruskal–Wallis test for each measurement category (model), only two out of the fifteen measurements showed statistically significant differences between the material groups: vestibular-oral and mesial-distal width. Post hoc analysis showed that the alginate model (mean range = 29.13) had significantly higher bias scores than the addition silicone model (mean range = 16.75) (z = 2.501, *p* = 0.037). The difference between the average values of the model bias made from condensation-based silicone and addition-based silicone was initially significant, but after applying the Bonferroni correction for further comparisons, this relationship did not remain significant (z = 2.197, *p* = 0.084). Addition-based silicone models had the highest accuracy in terms of fidelity, followed by condensation-based silicones, and then by alginate models. Silicone-based impression materials improved over time, being highly used in all areas of dentistry. Alginate impressions had the lowest pattern of accuracy among those studied.

## 1. Introduction

Impression materials are used to record and reproduce the exact morphology of the patient’s oral cavity. The impression is the negative replica of the dental field, and its accuracy depends on several factors. The most important factors are the impression technique and the materials used [1]. The result of a precise dental model is obtained through a correct impression technique and the use of impression materials with optimal properties. The doctor has the responsibility to have the knowledge about the dental materials and make the right determination in their use in the patient’s oral cavity. The quality of dental materials has evolved over time, along with improvements in applied techniques. The emergence of a new dental material takes place only after testing it on the basis of international standards in the form of rules given by certain competent forums such as FDI (International Dental Federation), ISO (International Organization for Standards), and DIN (German Institute of Standards). Compliance with these standards is aimed at launching dental materials on the market to ensure the ease of dental treatment and the safety of patients [2]. 

The dimensional stability of a material is its ability to maintain the accuracy of recording the details of the oral cavity for a longer period of time, including the time during imprinting and immediately after. This property is time dependent, so the best dimensional accuracy is immediately after the polymerization is complete, gradually decreasing until the time of mold casting [3]. The elastic memory of the impression material allows the return to the original dimensions, without creating distortions after the impression is disinserted from the oral cavity [4].

Studies have shown that polyvinyl siloxanes (PVS) have the best elastic memory (over 99%), so immediately after preparing the material, silicones (PVS) should be applied promptly and immediately after mixing [4,5]. On the other hand, polyethers retain their elasticity longer after preparation, but their stiffness at disinsertion is higher than that of silicones. Thus, the more flexible a material is, the easier it will be to disassemble and manipulate the impression. Studies have shown that polymers are the most rigid materials after setting, and the rigidity of silicones is dependent on the viscosity of the material [5].

Traditionally, impression material is classified by composition, polymerization reaction, and setting time. However, the most common way to categorize it depends on the properties of the material after setting [6].

Depending on the properties of the materials after they have set, they are commonly classified into rigid and semi-rigid (less used today), and elastic and synthetic elastomers [5]. The most commonly used orthodontic impression materials are irreversible hydrocolloids (alginates) due to their low cost, leading to accurate impressions in most cases. Other materials of choice are addition and condensation silicones, which have a higher accuracy and are used especially in situations with retentive areas, dental maxillary anomalies with crowding, or exaggerated malpositions. These are situations that require a higher accuracy of the study model.

Condensation silicones are materials often used today in dental practice due to their good properties. The polymerization reaction contains a reaction of functional tri- and tetra-alkyl silicates in the presence of tin octoate as a catalyst [3]. The material is formed after bonding between the end groups of the silicone polymer and the alkyl silicate in order to form a three-dimensional network with ethyl alcohol as a by-product. Subsequent evaporation may cause contraction after the completion of setting [7].

Polyvinyl siloxanes (PVS), also called addition-reaction silicones, are composed of two pastes that react by releasing hydrogen in the form of gas due to the interaction between moisture and the constituents of the polymer, without any by-products. This places them at the top of dental materials in terms of dimensional stability [8]. The viscosity of the addition silicones is dependent on the amount of silica filler, resulting in high viscosity (putty), medium, or low viscosity (fluids) silicones. Addition silicones require small amounts of catalysts to initiate a polymerization reaction. Anything that interferes with the catalysts will make the imprint sticky. Sulfur is usually the one that interferes, especially if latex gloves are used to handle the material.

The aim of this study was to evaluate the accuracy of three different impression materials commonly used in the dental practice with the aid of an extra-oral three-dimensional (3D) scanner using an in vitro analysis.

## 2. Materials and Methods

For this study, a typodont tooth model (Frasaco Gmbh, Tettnang, Germany) of the maxillary dental arch and mandibular dental arch (containing 16 permanent teeth) was used. 

**Figure 1:**
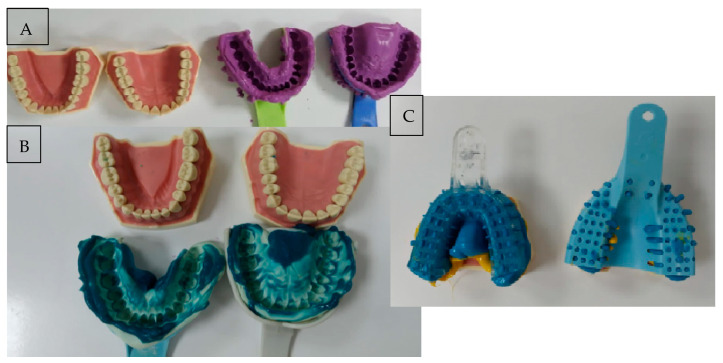
Impressions taken during the study using 3 different materials: (**A**) Alginate; (**B**) Condensing silicone; (**C**) Addition silicone.

With the aid of three different impression materials (Figure 1), this model was imprinted fifteen times resulting in fifteen different plaster models as follows:

○ Five plaster models were obtained using an alginate impression–generally named Model 1 to avoid any commercial issues;
○ Five plaster models were obtained using a condensing silicone impression–generally named Model 2 to avoid any commercial issues;
○ Five plaster models were obtained using an addition silicone impression–generally named Model 3 to avoid any commercial issues.

Later in the study, an GOM ATOS (Zeiss™ Gmbh, Braunschweig, Germany) capsule extra-oral scanner device was used to digitalize the models. This optical metering computer device uses two 12 MP CCD cameras along with a blue light LED projector to digitize the surface. Unencoded markers were used for spatial referencing of scans. The standard measurement deviation for reference markers is 3–5 µm (GOM, 2021). The acquired point cloud was then polygonized and transformed into a 3D mesh model. The scan of the reference model was used as a benchmark for comparison later in the study.

**Figure 2:**
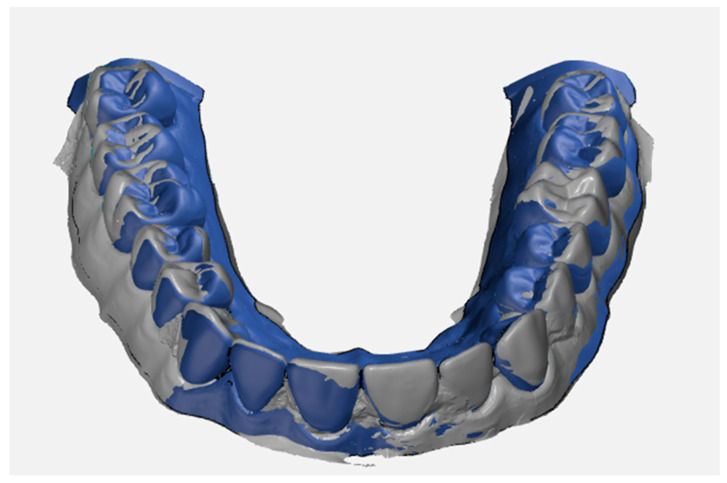
Over-imposition between reference model and plaster model.

**Figure 3:**
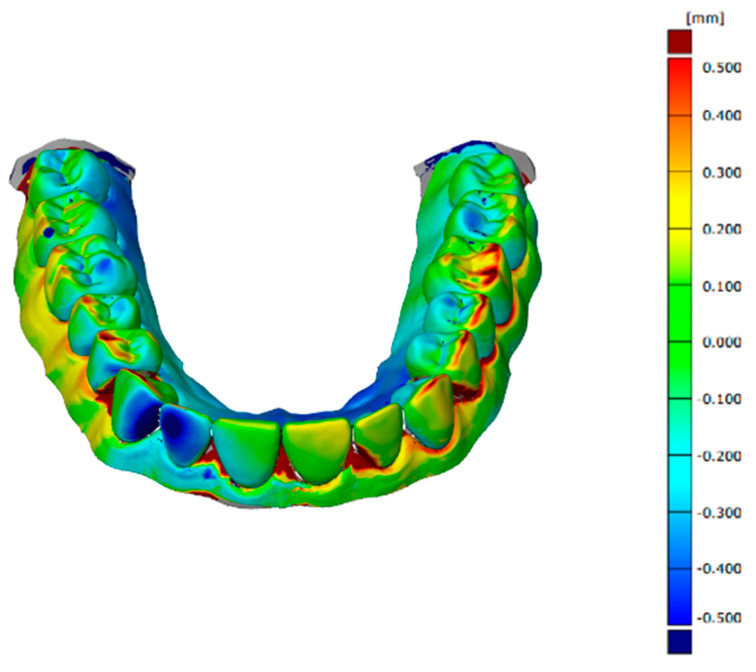
Deviations between the two surfaces when comparing the reference model to the alginate model.

**Figure 4:**
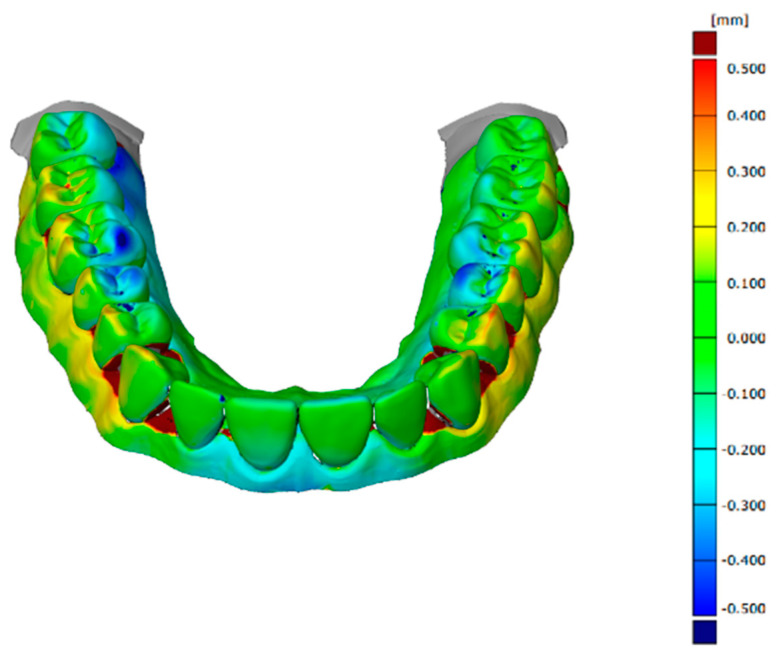
Deviations between the two surfaces when comparing the reference model to the condensation silicone model.

**Figure 5:**
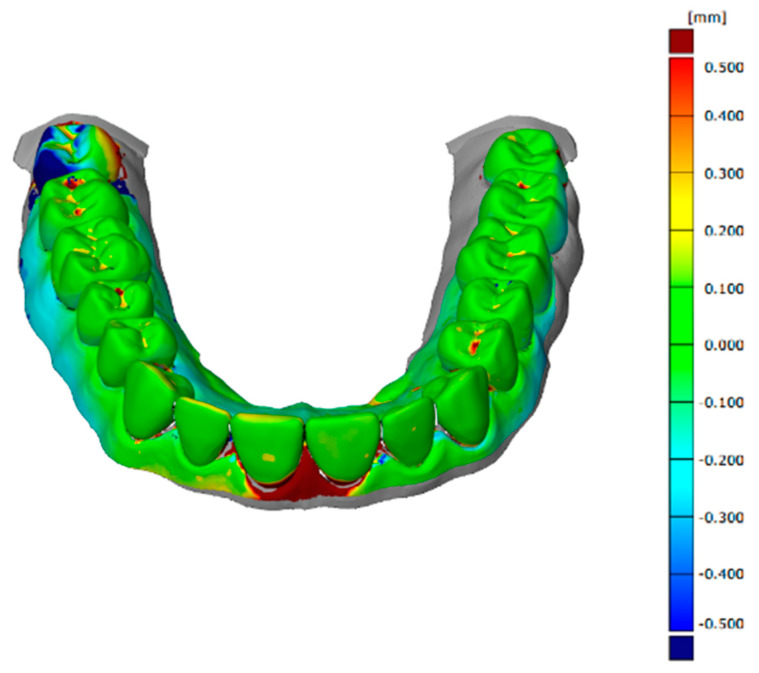
Deviations between the two surfaces when comparing the reference model to the addition silicone model.

The software used to align, compare, and measure scanned model surfaces was GOM Inspect ver. 2020. This is an inspection software used for measurements on mesh models, resulting from the processing of data from optical scanners. After pre-alignment, the reference scan (the reference model generated by the GOM ATOS software, version 6, Nikon Techmologies, Minato City, Japan) and the gypsum model scan were aligned using the local optimal matching method (Figure 2). This method aligns the two surfaces so that the average deviation between the two is minimal. Only the mesh portions containing the region of interest have been retained for good alignment. The deviations between the two surfaces of each material can be seen in Figure 3, Figure 4 and Figure 5. Regions presented in shades of yellow, orange, and red have deviations above the reference, while regions with shades of blue are below the reference.

**Figure 6:**
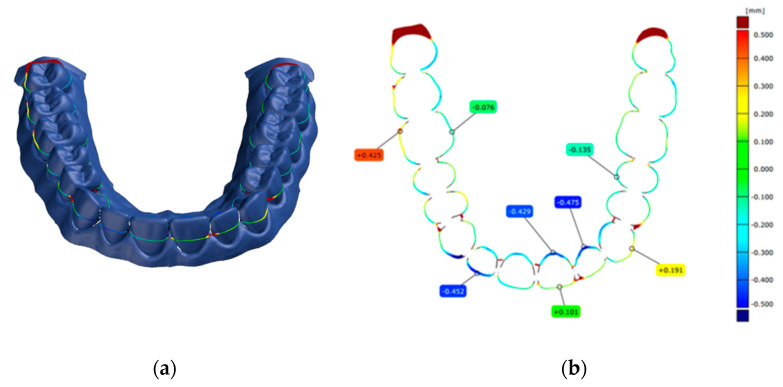
Section plans used for planned measurements (**a**) and measurements performed during the study (**b**).

For each plaster model studied (Model 1, Model 2 and Model 3), a section was created to perform the measurements (Figure 6). The section plan was created using the midpoints of the heights of the second maxillary right molars, second maxillary left molar, and the right maxillary central incisor. The height of each tooth was determined as the distance from the mucogingival junction to the occlusal surface.

**Figure 7:**
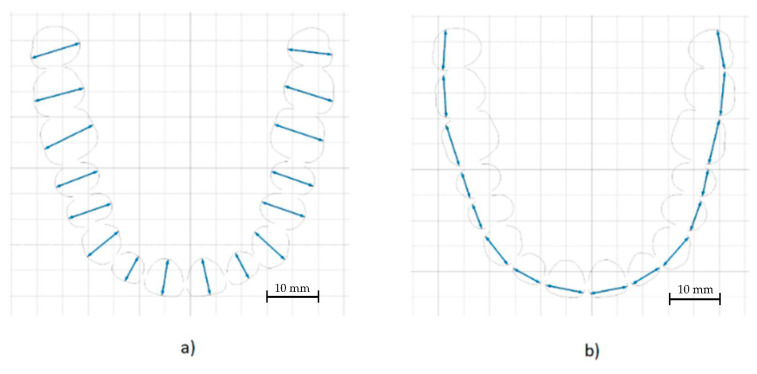

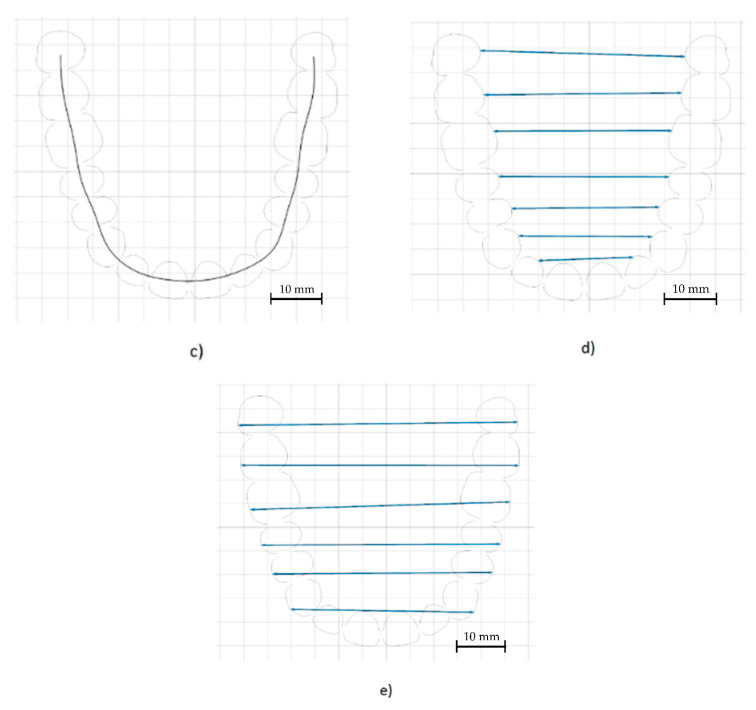
(**a**) Vestibular-oral measurement performed during the study. (**b**) Mesial-distal measurement performed during the study. (**c**) Curvature of the dental arch measurement performed during the study. (**d**) Inner arch length measurement performed during the study. (**e**) External arch length measurement performed during the study.

The measurements that were taken for each model were: the length of the arch curve, the mesiodistal width, the vestibular-oral width, and the lengths of the inner and outer arches. The vestibular-oral distance was measured by placing dots on opposite sides of each tooth in the vestibular-oral direction (Figure 7a). Repeatability was ensured by using the same measurement software for all models and applying the same technique. The mesial-distal width was measured in a similar way by placing the points along the teeth in the mesial-distal direction (Figure 7b). The curvature length of the dental arch was obtained by drawing a curve through the middle of the vestibular-oral distances (Figure 7c). The distances of the inner arch were determined by measuring the distances between the points used for the vestibular-oral widths placed inside the dental arch (Figure 7d). The distances of the outer arch were determined by measuring the distances between the points used for the vestibular-oral widths placed on the outside of the dental arch (Figure 7e).

*Bias* was used to evaluate the models. The bias is the absolute value of the deviation of the real values from the nominal values:

(1) $$Bias=abs\left(x_{actual}-x_{nominal}\right)$$

Statistical bias is a characteristic of a statistical technique or its results, whereby the expected value of the results differs from the true underlying quantitative parameter that is estimated. The particularity (*Bias*) of an estimator is the difference between the estimated value of an estimator and the actual value of the parameter that is estimated.

The following null hypotheses (H0) were tested:

**H0a.**  *There is no statistically significant difference between the buccal-lingual widths of the 3 types of models made of alginate, condensing silicone, and addition silicone.*

**H0b.**  *There is no statistically significant difference between the mesial-distal widths of the models made of alginate, condensing silicone, and addition silicone.*

**H0c.**  *There is no statistically significant difference between the distances of the inner spring of the models made of alginate, condensing silicone, and addition silicone.*

**H0d.**  *There is no statistically significant difference between the outer arch distances of the models made of alginate, condensing silicone, and addition silicone.*

The statistical processing of the obtained data was performed using the statistical software IBM SPSS v26, GraphPad Prism v6, and Microsoft Excel 365. The normality of the measurement distributions of the 3 models was evaluated using the Kolmogorov–Smirnov and Shapiro–Wilk normality tests. The Kruskal–Wallis test for independent data was used to compare the average bias values between the cementitious material groups. This test returns a significant result if there is at least one significant difference between a pair of groups. In cases of significant results, the procedure of Dunn (1964) [9] was used with a Bonferroni correction for multiple comparisons [10]. The chosen significance level was α = 0.05, *p* being considered significant when *p* < 0.05.

## 3. Results 

The mean (95% confidence interval) and standard deviations for bias in each measurement category are summarized in Table 1, where:

Model 1 = Model made using alginate;

Model 2 = Model made using condensing silicone;

Model 3 = Model made using addition silicone.

For the length of the arch, singular measurements were performed; therefore, no mean or standard deviation was calculated.

The distributions for each measurement model were tested for normality. Some distributions turned out to be normally distributed, while others did not. Aberrant values were identified in some samples. We decided to continue the analyses using non-parametric tests, as they do not make assumptions about data distribution and are less sensitive to aberrant values.

After performing the Kruskal–Wallis test for each measurement category (model), only two measurements showed statistically significant differences between the material groups: vestibular-oral and mesial-distal width (Table 2). Value distributions were not similar for all groups as assessed by visual inspection of a box chart. Average ranks were reported along with adjusted *p* values.

The test to determine whether there were differences in bias values for vestibular-oral width between different groups of materials: alginate (Model 1), condensing silicone (Model 2), and addition silicone (Model 3) showed that the median values were statistically significantly different between the different groups of materials (H (2) = 9600, *p* = 0.008). Subsequently, the pair/dependent comparisons showed that the model produced from alginate (average range = 32.97) had statistically significantly higher scores than the model produced from addition silicone (average range = 18.03) (z = 2501, *p* = 0.008).

This was also true for mesial-distal width, where statistically significant differences were found between the averages of the alginate, condensing silicone, and addition silicone material groups (H (2) = 7449, *p* = 0.024). Post hoc analysis showed that the alginate model (mean range = 29.13) had significantly higher bias scores than the addition silicone model (mean range = 16.75) (z = 2.501, *p* = 0.037). The difference between the average values of the model bias made from condensing silicone and addition silicone were initially significant. However, after applying the Bonferroni correction for multiple comparisons, the relationship did not remain significant (z = 2.197, *p* = 0.084).

**Figure 8:**
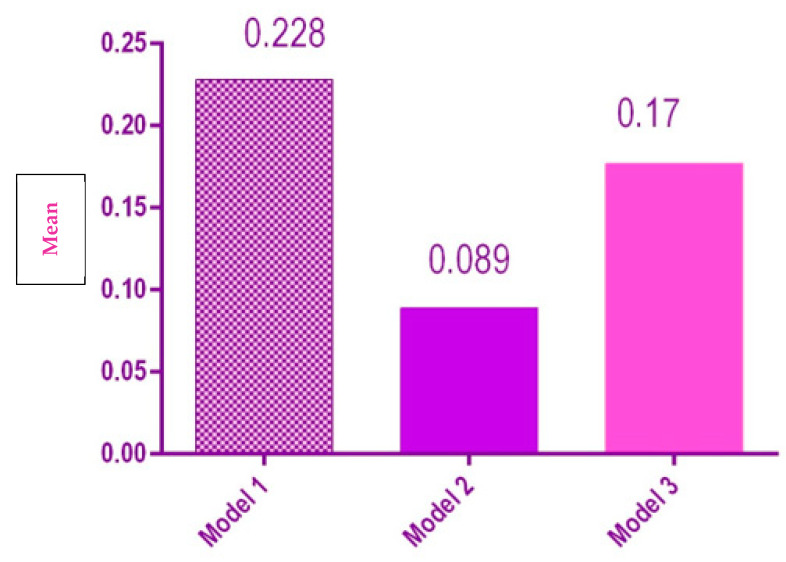
Statistical results of the length of the dental arch for the 3 models.

There was no statistical significance difference when comparing the means of the arch length for the three models (Figure 8).

**Figure 9:**
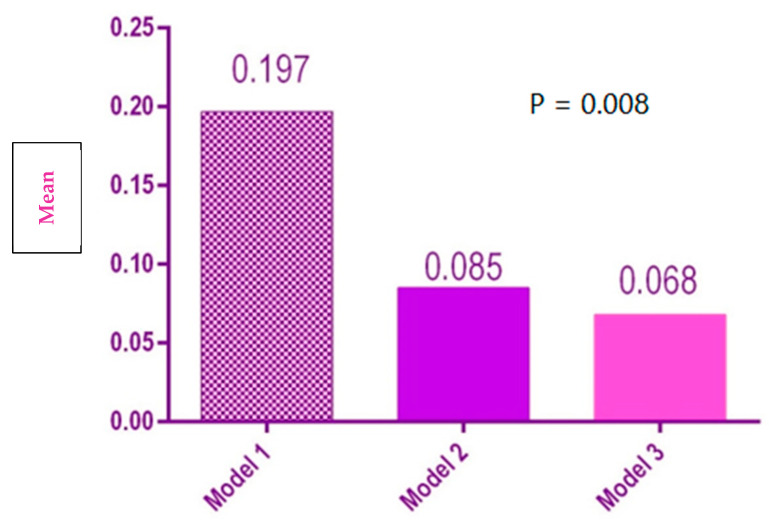
Statistical results of vestibular-oral distances for the 3 models.

Model 1 had a significantly larger vestibular-oral width than Model 2 and Model 3 (*p* = 0.008) (Figure 9).

**Figure 10:**
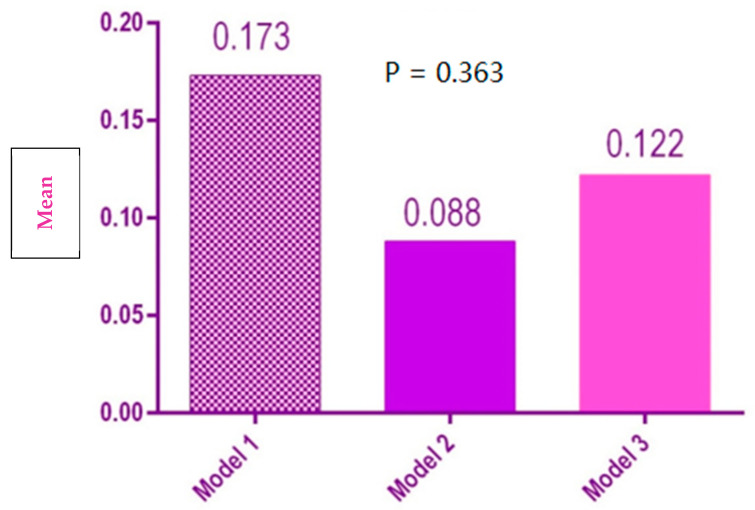
Statistical results of the inner arch distances of the 3 models.

There was no significant difference between the interior distances for the three models (Figure 10).

**Figure 11:**
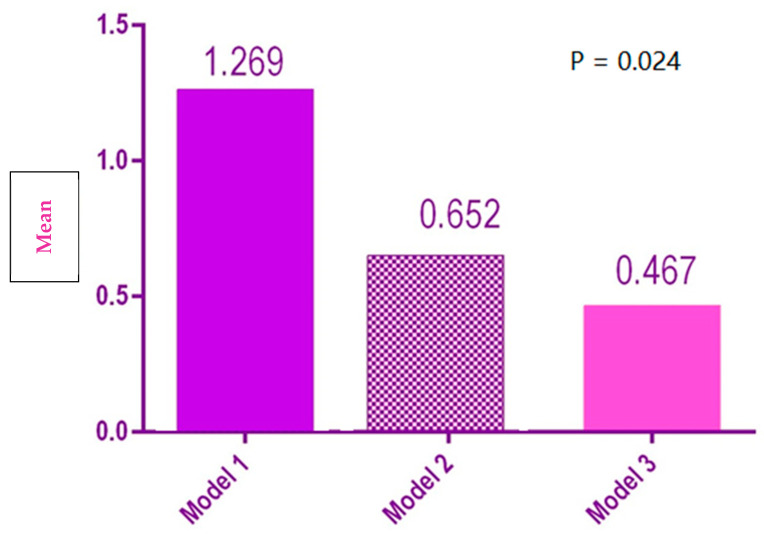
Statistical results of mesial-distal distances for the 3 models.

There was a significant difference between the mesial-distal width of the three groups, Model 1 having the mean mesial-distal width that was significantly higher than Model 2 and Model 3 (Figure 11).

**Figure 12:**
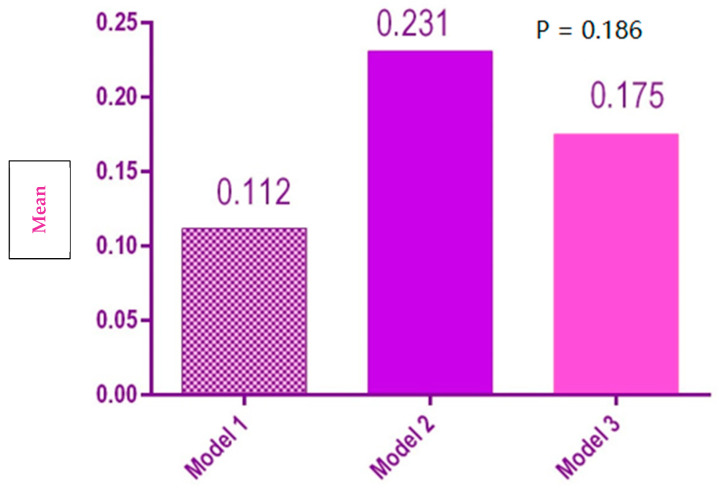
Statistical results of the external distances of the dental arch for the 3 models.

There was no significant difference between the three models regarding the average of the external distances of the dental arch Figure 12).

## 4. Discussions

In our study, the plaster models obtained with the aid of alginate impression had the lowest accuracy in terms of dimensional stability, especially when measuring the vestibular-oral and mesial-distal width compared with the reference models. Our results are consistent with Peutzfeld et al. [11] who showed that alginate has the lowest accuracy (160 ± 19 µm). It was also reported that the timing of casting the model after the impression has an extremely important role, as well as the way in which the impression is disinfected. Furthermore, Torassian et al. [12] showed in their study that the dimensional stability of any impression material is time and technique dependent. They showed that some materials have good dimensional stability only immediately after imprinting, which decreases significantly after a few hours. The most important change was observed in the case of alginate (36.93 at imprinting, 36.21 at 72 h, 36.44 at 120 h, and reaching 36.40 after a week). 

Further studies that evaluated impression materials revealed that the major disadvantage of alginates is their poor dimensional stability. This is due to the phenomena of imbibition and syneresis that occur [13]. Therefore, it is advisable to pour them within 10–12 min to avoid distortion. Their breaking strength is low, breaking easily in retentive areas, such as the subgingival space or areas with dental crowding. Alginate powder containing silicon, lead, or cadmium has been tested as toxic. This led to a more extensive study of these materials. Following research and efforts to improve their quality proved that it was possible to delay the casting of the models by up to one to four weeks [14]. Bubble defects on the occlusal faces of the teeth recorded on the model after casting alginate fingerprints are due to the incorporation of air or saliva bubbles between the material and the occlusal surface of the teeth. A study by Cervino et al., in 2018, demonstrated that smoothing the material with a damp finger and rinsing the mouth before the impression would give better results [15].

Further studies that evaluated silicone impression materials evaluated the hydrophobicity of these materials, which is due to the surface formed by chains of paraffinic methyl groups. This disadvantage can compromise the result by encapsulating saliva or blood particles if the field has not been well-dried and prepared [8]. Attempts have been made to improve them by adding non-ionic surfactants. However, it has been found that these substances come off the surface of silicones during exposure under continuous water jet or during arch disinfection. The alcohol released during the polymerization reaction is a plasticizer, being a small mobile molecule [16]. This by-product will eventually lead to the shrinkage of the material over time due to evaporation from the surface. Permanent deformity is about 1–3%, which is quite large. The breaking strength is lower than that of poly-sulfur elastomers (3000 g/cm) [12]. On the other hand, under the microscope, Vitti et al. [7] analyzed the accuracy of several silicone-based addition and condensation materials from different manufacturing companies with impressions being taken in several ways. Their results showed that the condensation silicones had the most notable differences in terms of dimensional stability, while the addition silicones did not have statistically significant differences (*p* > 0.5). At the same time, the study showed that the impression technique did not influence the accuracy of the materials [7]. Their results are not consistent with our findings.

In 2019, Martins evaluated the dimensional changes in the first VPS impression type. The material shrinkage was 0.29% ± 0.15% after setting, 0.32% ± 0.21% at 24 h, and 0.30% ± 0.23% after 1 week. No significant shrinkage of the silicone was found over time; the silicone can be stored for a week without the risk of clinically significant dimensional changes and is a reliable and affordable replacement for alginate [8].

In 2014, Sinobad et al. showed that Elite HD + Regular body silicone (Zhermack, Badia Polesine, Italy) had a 0.16% dimensional change immediately after setting, which increased to 0.40% after 24 and to 0.52% seven days later. This tells us that the tested silicone showed good results though being marketed as a preliminary impression material [17].

Impression technique is a critical variable in the accuracy of VPS impressions, but Nouri et al., in 2019, concluded that custom trays do not significantly increase the accuracy of impressions, and rigid stock trays are suitable alternatives [18].

In a literature review, Naumovski, in 2017, confirmed the lack of standardization of the methodologies applied in the research of the dimensional stability and accuracy of silicone-based impression materials, but all the findings point to the superiority of the addition silicone compared to the condensation silicone in all parameters, as well as to other elastomers [19].

Lee in 2022 stated that digital workflows in dentistry have improved the clinical experience for both patients and clinicians, but the accuracy of digital workflows is not always superior or equivalent to that of their conventional analogs, including the impression techniques [20].

Kim’s results showed that the laser scanned models are highly accurate compared to plaster models and CBCT scans, so laser scanners and laser printers are used for the best results [21].

The models measured manually and digitally showed certain similarities, but the use of digital models offer advantages to the orthodontists (easy storage, saves time and space) but we still have to consider the element of cost [22].

To combat one of the possible disadvantages of these materials (which is the hydrophobicity and thus the need for a dry field to accurately record the details), manufacturers have incorporated surfactants into their composition and produced hydrophilic addition silicones [23]. Another alternative would be to introduce polyether chains. They have an intrinsic hydrophilicity which, diluted by silicone, would make the silicone more hydrophilic [24]. The main disadvantage of this option is the higher water absorption and the possible separation of some chemical phases [12]. This technique has other disadvantages, such as the considerable waste of raw material and the accuracy of the dental models produced being dependent on the size of the cutting burs of the milling machine. Frequent wear of the milling burs also occurs during the production of large objects as the dental models [25,26]. Even so, the newly manufactured and improved silicone addition materials have viscous–elastic behavior when all precautions are taken in their use. 

Very recently, Liu et al. (2022) investigated the geometric accuracy of 3D-printed dental implants for biomedical applications and concluded that this technique is still in its infancy. More research and clinical studies need to be carried out to establish the methodologies of 3D printing techniques and understand the long-term safety and clinical efficacy of 3D-printed implants [27,28].

## 5. Limitations of the Study

This study has limitations, such as the small number of study models. Another possible limitation of the present study is due to the in vitro evaluation which is different for the oral cavity environment and can influence the results. The present study contributes to the increase in reference data in the literature, supporting future research in this field.

## 6. Conclusions

Our results highlight that the addition silicone models have the highest accuracy in terms of fidelity, followed by the condensation silicones and then by the alginate models. These findings confirm the results of previous studies performed on impression materials. Silicone-based impression materials have improved over time, being highly used in all areas of dentistry. Alginate impression may only be an option if high-pattern accuracy of the model is not required, as it has the lowest accuracy among the impression materials studied.

## Figures and Tables

**Table 1:** Descriptive statistical indicators of the characteristics for the three models.

Distance	Model nr.	*Bias*	
		Mean [95% CI]	Standard Deviation
Arch length	1	0.228	-
	2	0.089	-
	3	0.177	-
Vestibular-oral width	1	0.197 [0.104, 0.291]	0.176
	2	0.085 [0.053, 0.116]	0.06
	3	0.068 [0.037, 0.1]	0.059
Mesial-distal width	1	1.269 [0.273, 2.264]	1.869
	2	0.652 [0.267, 1.038]	0.724
	3	0.467 [−0.312, 1.245]	1.461
Inner arch length	1	0.173 [0.044, 0.301]	0.139
	2	0.088 [−0.018, 0.193]	0.114
	3	0.122 [−0.038, 0.282]	0.173
External arch length	1	0.112 [−0.008, 0.231]	0.114
	2	0.231 [0.137, 0.324]	0.089
	3	0.175 [−0.02, 0.37]	0.186

**Table 2:** Hypothesis testing results of the study.

Distance	Statistic (H)	*p* Value	Null Hypothesis H0
Vestibular-oral width	9.600	0.008	Rejected
Mesial-distal width	7.449	0.024	Rejected
Inner arch length	2.026	0.363	Accepted
External arch length	3.934	0.140	Accepted

## Data Availability

All data regarding this manuscript can be checked with the corresponding author at alexandru.vlasa@umfst.ro.
